# Access to health and rehabilitation services for persons with disabilities in Sierra Leone – focus group discussions with stakeholders

**DOI:** 10.1186/s12913-022-08366-8

**Published:** 2022-08-05

**Authors:** Lina Magnusson, Ismaila Kebbie, Victoria Jerwanska

**Affiliations:** 1 Department of Health Sciences, Faculty of Medicine, Lund University, Postal Address Box 157, 221 00 Lund, Sweden; 2 National Rehabilitation Centre, Ministry of Health and Sanitation, Sir Samuel Lewis Road, Murray Town, Freetown, Sierra Leone

**Keywords:** Disability, Focus group discussions, Health systems, Rehabilitation, West Africa

## Abstract

**Background:**

In Sierra Leone persons with disabilities are at higher risk of living in poverty and have poor access to a fragile healthcare and rehabilitation services. The aim was to explore stakeholders’ perceptions of access to health and rehabilitation services for persons with disabilities in Sierra Leone.

**Methods:**

Seven focus group discussions, including stakeholders working within the field of disability was conducted.

**Results:**

The subthemes were: continuous stigmatisation of persons with disabilities throughout life; long distances and transportation issues to access health and rehabilitation facilities; financial constraints; infrastructural barriers to healthcare and rehabilitation services and healthcare personnel’s negative attitudes and inadequate knowledge towards persons with disabilities; rehabilitation and healthcare facilities lacking materials to provide quality services; lack of specialised services and rehabilitation personnel for complex rehabilitation and the need for continuous education of new and current rehabilitation personnel.

**Conclusion:**

Local actors need to take charge and renew efforts made by international organisations by providing trained rehabilitation staff and quality rehabilitation services. Rehabilitation services need to be affordable and transportation costs covered for persons with disabilities to access healthcare and rehabilitation services. Continuous education of the public and health personnel about disability is necessary to reduce negative attitudes towards persons with disabilities.

## Introduction

Persons with disabilities (PWD) are at higher risk of living in poverty, being unemployed, and having less access to healthcare compared to the general population [1]. Sierra Leone is a English speaking low-income country located in West Africa, with approximately 57% of the population living in poverty and 13% in extreme poverty [2]. Sierra Leone has a fragile healthcare system. Between 1991–2002 Sierra Leone suffered a civil war with polio outbreaks [3, 4] and between 2014–2016, an Ebola virus outbreak destroyed much of the country’s health and rehabilitation infrastructure [5, 6]. The country has a double burden of disease, with a high prevalence of and mortality rates due to communicable diseases. Non-communicable diseases and injuries are also increasing [6]. The prevalence of disability in Sierra Leone is 4.3%, according to the 2018 integrated household survey [2]. According to the survey, the most prevalent types of disability were lower limb impairments (32%), visual impairments (29%), and hearing impairments (9%). The main causes of disability were diseases or illness (42%), followed by congenital causes (17%) [2]. PWD need general healthcare like the rest of the population and specialised healthcare related to the health condition that caused the impairment [1]. PWD also need access to rehabilitation, including rehabilitation medicine, therapy and assistive technology because it increases the possibility to achieve and maintain optimal functioning in interaction with their environments which provides possibilities for greater participation in society [1].

Sierra Leone has signed the declaration of the Convention on the Rights of Persons Living with Disabilities (CRPD) [7]. Article 25 and 26 of the CRPD [8] identifies healthcare and rehabilitation as human rights for PWD. The Government of Sierra Leone has adopted the CRPD by implementing The Disability Act [7], which states that PWD are entitled to free healthcare in public healthcare facilities. However, Sierra Leone’s Free Health Care Initiative [9] is aimed at specific vulnerable groups, such as pregnant women, lactating mothers, and children under five, and PWD access to the Free Health Care Initiative has been unclear. However, the 2018 integrated household survey indicated that PWD to some extent had access to this programme [2]. To enable implementation and enactment of policies to provide healthcare, rehabilitation, and assistive technology for PWD, multisectoral stakeholders engagement and collaboration is necessary [5], for example, between stakeholders from different ministries, international bodies, non-governmental organisations (NGOs), PWD and health/rehabilitation personnel.

Most PWD in Sierra Leone live in poverty [2], have poor access to healthcare services, rehabilitation or mobility devices such as prostheses and orthoses [10, 11]. PWD also have less access to education and employment than those without disabilities [2, 10]. In Sierra Leone, there is a need to improve access to rehabilitation services and knowledge regarding this. To our knowledge, there is no previous study in Sierra Leone investigating access to rehabilitation from a stakeholder’s perspective working within health, rehabilitation, and disability organisations. The aim of this study is to explore stakeholders’ perceptions of access to health and rehabilitation services for PWD in Sierra Leone.

## Method

### Design

A qualitative study using focus group discussions (FGD) with stakeholders working in the field of disability in Sierra Leone [12] was applied. FGD was chosen to capture discussions, reasoning, and suggestions from different stakeholders’ perspectives [12]. Content analysis was applied to the data [13]. Two content areas were discussed in the FGDs: access to healthcare and rehabilitation, which is covered in this paper, and coordination of health and rehabilitation, which is presented in a separate paper [14]. Persons with physical disabilities were interviewed about their experiences accessing and using rehabilitation and healthcare services in another part of the project [11].

### Procedures 

The questioning route developed according to Kruger [12] covered; opening questions *“Could you please tell us your name, what type of organisation you work for and how long you have been working within health or rehabilitation services?* and introductory questions *“Could you describe what you work with within health and rehabilitation services?”*. Examples of key questions were *“In your experience what obstacles do people with disability face in accessing healthcare services? Do you have suggestions on what could be done to improve access for people with disability to health services? If you could change one thing to improve access to rehabilitation services provided for people with disability today, what would you change?”* The discussion was summarised by the moderator, followed by an ending question “*Is there anything that you feel that we missed and would like to add?*”. The questioning route was developed by authors Lina Magnusson (LM) and Victoria Jerwanska (VJ) and validated by staff at Humanity and Inclusion, Sierra Leone. Probing questions were used throughout the FGDs. Ethical approval was obtained from the National Scientific Ethical Review Committee in Sierra Leone [15].

A purposive sampling was applied with the aim to achieve variation of stakeholders working within the field of disability, including, government officials, policy makers, local healthcare and rehabilitation staff, international experts working for NGOs and representatives of disability organisations, in Sierra Leone. Inclusion criteria were stakeholders English speaking, and above 18 years of age working within health for PWD, rehabilitation, or disability issues. All stakeholders working within health for PWD, rehabilitation, or disability issues in Freetown, Makeni and Bo were invited to participate in the study. In Freetown invitations to participate in a FGD were administrated through Ministry of Health and Ministry of Social Welfare, the National Commission for Persons with Disabilities, Humanity and Inclusion, the Physiotherapy Department at Connaught Hospital, the Physiotherapy Department at Emergency Hospital, Sightsavers, the National Rehabilitation Centre, World Hope International Sierra Leone, and Sierra Leone Union on Disability Issues. In Makeni, invitations were also administrated through the management of the Rehabilitation Department at Makeni Government Hospital and in Bo to Bo Regional Rehabilitation Centre to achieve a geographical variation.

Seven FGDs were conducted in March 2019, four in Freetown, the capital city; one in Makeni, in the northern district; and one in Bo, in the southern district. The FGDs were moderated by the authors (VJ), (LM) and trained co-moderators. The questioning route was covered in all FGDs to contribute to increase trustworthiness of data. The FGDs were recorded using a digital voice recorder which lasted an average of 74 min, (range: 58–91 min). All FGDs were transcribed verbatim by author (VJ) and generated rich qualitative data material consisting of 127 pages of text.

### Data analysis

The data analysis was conducted by authors LM and VJ using content analysis [13]. Transcripts were read several times to get an overview of the content. The data relating to the content of access to health and rehabilitation services were analysed for this paper. First, transcripts were divided into meaning units, e.g. sentences or paragraphs related to the study’s purpose and different to each other [13]. The data material was rich and saturated [16]. Next, each meaning unit was condensed, which is to shorten without losing its meaning. Each condensed meaning unit was then assigned a code which is an abstracted label to the core content of the meaning unit, Table 1. Finally, codes were sorted into subthemes and themes [13]. The body of results text represents all the codes and are exemplified with quotes. Investigator triangulation [16] refers to the use of multiple researchers to make coding, analysis and interpretation decisions. Author LM and VJ worked together with data anlysis to ensure different perspectives were discussed and increase credibility. Author Ismaila Kebbie (IK) reviewed the result for cultural validity and contributed to interpreting the result. The sound files and transcripts were stored safely.

**Table 1:** Example of meaning units, condensation, code, and subtheme

Meaning unit	Condensed meaning unit	Code	Subtheme
*“That is the reason, because if you do not have money to pay. So, they pay less attention to us. Those who pay money, they give them great attention, because they pay them money… But for PWD even though it is already [been] announced that the PWD should have free care at all health departments. They still have a problem to access the facility. They are looking for money, they just want money…that is the one reason”.* FGD B, Participant 8, certified occupational therapist, governmental organisation	If you do not have money the health and rehabilitation staff they pay less attention. Those who pay, they give great attention. It is announced that PWD should have free healthcare at all departments but still they have problems accessing the facilities without money	Personnel did not attend to PWD in healthcare facilities due to not getting money from them	Healthcare personnel’s negative attitudes and inadequate knowledge towards persons with disabilities
*“We also have this financial aspect, most of us PWD are not working. We do not earn salary monthly. So, the cost because for now, the devices we no longer have it free of cost, we must pay for it. Like for instance, the one I am using now I pay 250,000 Leones. So not all persons with disability will afford to pay for these devices or other services. So, you can find out most of them they go without devices”.* FGD F, Participant 28, disability advocate, non-governmental organisation	We have financial aspect most are not working and do not earn a monthly salary. The assistive devices are no longer free of charge, we must pay. The one I am using cost 250,000 Leones (about 20US $).PWD cannot afford to pay for devices and go without	Having to pay for assistive devices led to many PWD living without assistive devices	Financial constraints

### Self-reflexivity

In qualitative research, the researcher is an instrument, making active decisions during the research process [17]. Therefore researchers pre-understandings influence the research process [18], especially in cross-cultural research settings [17]. Two authors are Swedish, LM with a background in disability research, public health, and prosthetic and orthotic services, and, VJ in public health and nursing. LM has extensive previous experience in qualitative research and collecting data in Sierra Leone and other low resource settings. Author IK is a senior physiotherapist from Sierra Leone with a public health background, working within rehabilitation service delivery from both clinical and policy perspectives.

## Results

The seven FGDs consisted of 37 participants, with 4–7 participants in each focus group, Table 2. The average age was 41 years (range: 21–55 years). Their average experience within the field of disability, rehabilitation and healthcare services was 13 years (range: 1 month-36 years). All participants were literate, the visually impaired participant read and wrote brail. Most participants were educated with an average of 14 years in school (range: 0–18 years).

**Table 2:** Participants demographics and characteristics, *N* = 37

	**Study population n (%)**
**Sex**	
Male	24 (65)
Female	13 (35)
**Occupation**	
Health and Rehabilitation	
Certified prosthetics and orthotic technician	6 (16)
Nurse (BSc or below	4 (11)
Certified Physiotherapist	2 (5)
Certified rehabilitation therapist	2 (5)
Certified Occupational therapist	1 (3)
Clinical health officer	1 (3)
Diploma in rehabilitation medicine	1 (3)
Assistant physiotherapist	1 (3)
Other	
Disability advocate	5 (13)
Technical/Operational coordinator	4 (11)
Governance/Leadership/Policy expert	3 (8)
Administration/Business/Finance	2 (5)
Monitoring/Evaluation	1 (3)
Social worker	1 (3)
Teacher	1 (3)
Missing	2 (5)
**Organisations**	
Governmental organisation	21 (57)
Non-governmental organisations	9 (24)
Disabled persons organisations	7 (19)
**Country**	
Sierra Leone	31 (83)
England	1 (3)
France	1 (3)
Italy	1 (3)
Mexico	1 (3)
Norway	1 (3)
United States of America	1 (3)

Eight subthemes and two themes emerged, Table 3.

**Table 3:** Summary of themes and subthemes

Subthemes	Themes
Continuous stigmatisation of persons with disabilities throughout life Long distances and transportation issues to access health and rehabilitation facilities Financial constraints Infrastructural barriers to healthcare and rehabilitation services Healthcare personnel’s negative attitudes and inadequate knowledge towards persons with disabilities	Social, economic, and environmental impediments in accessing health and rehabilitation services
Rehabilitation and healthcare facilities lacking materials to provide quality services Lack of specialised services and rehabilitation personnel for complex rehabilitation Need for continuous education of new and current rehabilitation personnel	The necessity for improved health and rehabilitation service delivery for persons with disabilities

### Social, economic, and environmental impediments in accessing health and rehabilitation services

#### Continuous stigmatisation of persons with disabilities throughout life

The participants expressed the stigmatisation of PWD often begins in childhood by the family’s treatment. The participants perceived that in Sierra Leone many children with a disability tended not to live with their biological parents, and many were abandoned due to their disability. Parents and children with a disability were isolated and often lacked networks with other families in the same situation. Participants described PWD were sometimes not considered to be whole human beings, therefore not able to contribute and socialise in their community.

“Being a disabled in Sierra Leone is like being a half. People do not consider you, even the community, because you are just like a half. They do not recognise you, and they do not count on you. You can see some families like they will say, “I have three sons” if one of them have a disability, they will say, I have two and a half”. FGD D, Participant 21, assistant physiotherapist, governmental organisation.

Participants described that PWD often lacked opportunities to go to school, and their teachers perceived knowledge about disability was lacking. PWD that were educated still faced discrimination in seeking employment. Participants expressed that lack of knowledge and prejudice about disability in society decreased inclusion opportunities for PWD. Changing the mindset of society was seen by the participants as important to ensure equal access to opportunities. Sensitising communities on disability matters and human rights for PWD were also needed at all levels of society. Negative perceptions towards PWD impacted their self-esteem negatively. Participants described knowledge about medical conditions such as non-communicable diseases leading to disabilities was lacking in public and amongst healthcare personnel which created stigmatisation.

Participants disclosed that PWD did not access rehabilitation centres nor use the assistive device due to fear of people seeing and shaming them. They feared ill-treatment from rehabilitation personnel, although assistive devices could give them increased mobility opportunities. Spreading information about rehabilitation services through television and radio was perceived by the participants as a good way of reaching people. As was involving religious and community leaders to raise awareness. Participants discussed religious and community leaders should be regarded as key rehabilitation stakeholders because people trust and listen to them. They could therefore help motivate PWD to seek rehabilitation services.

**“**They [religious leaders] would be the ones like our own ambassadors to talk to the community. We make sure that we incorporate counsellors or the religious leaders in each community in anything we do. They are the ones who reach these people more than us and their voices are stronger than ours”. FGD E, Participant 26, nurse, governmental organisation.

#### Long distances and transportation issues to access health and rehabilitation facilities

Participants expressed concerns that rehabilitation service coverage was poor and missing in major referral hospitals. Long distances and inadequate transportation to rehabilitation facilities was a challenge experienced by PWD living in both rural and urban areas, which could negatively affect their health condition before arriving at the health facility. Participants described rehabilitation centres lacked funding and transportation to conduct outreach services. Participants described public transport drivers do not stop for PWD due to their slow pace in climbing into the vehicle.

“In Freetown it is much easier to access a vehicle. Thinking about those deep in the provinces, where even to access a vehicle is very difficult. Maybe they use this bike, and the bike rider may not have that patience enough to wait for disabled whose movement is so slow”. FGD C, Participant 12, administration/business, disabled persons organisations.

Participants suggested that disability adapted vehicles should be imported. PWD could not afford to pay for long-distance transportation, and they had to pay extra because of being charged for their wheelchair. To ensure access to rehabilitation facilities and assistive devices, participants suggested opening new rehabilitation centres to cover more districts, strengthening rehabilitation services by incorporating community-based rehabilitation (CBR) into peripheral health facilities and training community volunteers in combination with outreach programmes.

“Government is supporting and funding malaria programs and other programs on health. They provided a motorbike for them to do follow up in the community to make sure that things are going as things are supposed to. There are people who have problems in terms of rehabilitation that are living in the rural parts. For the rehabilitation department there are no follow up for people that we are producing devices to. Visit the patient, make sure that the patient is using the devices and how it is using this device. There is no mobility to give support to those activities”. FGD D, Participant 22, certified prosthetics and orthotic technician, governmental organisation.

#### Financial constraints

Participants explained that accessing healthcare and rehabilitation services was expensive due to PWD having to pay for registration, consultation fees and other services like transport within the hospital and drugs even though they should be provided for free. Due to high rates of unemployment, PWD had difficulties raising money to pay for their healthcare. Participants described NGOs used to provide assistive devices free of charge, but that changed when the rehabilitation centres were handed over to the Ministry of Health and Sanitation; PWDs started paying for assistive devices which led to the loss of mobility due to the cost. Some had to sell their belongings to afford an assistive device to meet their necessities. Participants expressed frustration over a lack of materials leading to persons with money being the only one’s able to access assistive devices.

“PWD, those that are in the deep rural areas, those that are in the villages for them to access a health centre it is very difficult. Even to get their daily living consumption is very difficult. Then to come here to the health centre, where they pay transportation from there to here. When they come, they [healthcare personnel] charge them for the material. Because the service is free but the materials which the personnel is using, they must pay for. Poverty is the thing that hinder them not to come to the health facility”. FGD B, Participant 7, certified rehabilitation therapist, governmental organisation.

Participants suggested decreasing the economic constraints for PWD could increase the utilisation of health and rehabilitation services. Participants called for aid to help the situation for PWD through financial assistance from the Ministry of Social Welfare, Gender and Children’s Affairs or the establishment of a National Disability Trust Fund.

#### Infrastructural barriers to healthcare and rehabilitation services

Participants expressed that accessibility for PWD was not taken into consideration when building public structures. Generally, public buildings, including healthcare and rehabilitation facilities, were not physically accessible for PWD. Participants described limited availability of ramps, escalators, rails and narrow doors caused missed opportunities more broadly in education and employment but also in accessing treatment from healthcare and rehabilitation facilities.

“The infrastructure is not disabled-friendly; it is the environment that is making us more disabled. We have nothing here; most hospitals do not have a ramp. So, it means accessibility is a challenge. You have to be dehumanised by [people] carrying you for you to be able to go to some of these hospitals”. FGD F, Participant 33, disability advocate, disabled persons organisations.

#### Healthcare personnel’s negative attitudes and inadequate knowledge towards persons with disabilities

Participants disclosed the free healthcare policy for PWD led to healthcare personnel not attending to PWD or treating them last because they could not or should not pay for the healthcare service. Participants expressed that PWD faced more stigmatisation from healthcare personnel than rehabilitation personnel. Healthcare professionals’ ignorance and lack of pride in working with PWD affected PWD’s care.

“We had an amputee that had a shoulder dislocation, we struggled to get anaesthetics so that they can fix this shoulder because of the pain. It took us two days, begging, begging. Not until one guy who is a student, I spoke to him, he came and helped us, and we fixed the shoulder. But if this is a normal person coming with shoulder dislocation, “Okay, come!” Because they are expecting a huge amount [of money] out of that patient”. FGD B, Participant 27, certified prosthetics and orthotic technician, governmental organisation.

Participants further described healthcare personnel displayed negative attitudes regarding sexual and reproductive health rights for PWD, for example, shaming pregnant women with a disability. Persons with hearing impairments lacked interpreters and faced discrimination by staff in healthcare facilities. Participants raised concerns that doctors, midwives, and nurses lacked experience, knowledge and understanding of working with PWD. Doctors and surgeons had a biomedical perspective on health. Still, they lacked knowledge and a holistic perspective which led, for example, to a person being amputated without a referral to rehabilitation services. Participants suggested the training curriculum for doctors, nurses and other healthcare professionals should include early detection and prevention of disability to ensure appropriate care for PWD and to understand the importance of rehabilitation.

“Lack of training [for healthcare workers] of different types of disabilities is an issue here that at least needs to be recognised. You may not know what to do with it but to understand that something is not right, so that something needs to be done. Just like you would do with clubfeet. You would recognise that a kid has clubfeet and then do something about it. Usually, it is the health workers that have the initial interaction with patients”. FGD A, Participant 2, teacher, non -governmental organisation.

### The necessity for improved health and rehabilitation service delivery for persons with disabilities

#### Rehabilitation and healthcare facilities lacking materials to provide quality services

Participants explained that although the Free Health Care Initiative had been proclaimed, healthcare centres had not gotten the support they needed to provide services free of charge. Participants described necessary healthcare materials and goods were not available. Rehabilitation centres´ machines, and other equipment needed replacements or maintenance to continue to provide assistive devices. Since the government took over services from NGOs rehabilitation centres lacked electricity and running water to produce assistive devices. Rehabilitation professionals got salaries, but the government provided no materials for assistive devices. Participants explained NGOs used to import the needed materials for assistive devices, pay for patient accommodation and provide vocational training for PWD, but this has stopped. Poor, recycled materials lead to PWD paying for low quality assistive devices. Sometimes PWD were asked to provide their own materials to get assistive devices made.

“I come in the morning for work, and then my duty is to discharge my responsibility. When my client comes, I would like to give him services, but I do not have the materials to produce assistive devices. How can I work for that client?” FGD D, Participant 22, certified prosthetics and orthotic technician, governmental organisation.

Participants expressed frustration seeing PWD lose their mobility as they could not provide or maintain assistive devices as before or knowing that PWD were paying for long-distance transportation to reach rehabilitation centres but would not get the anticipated help.

“When you go for orthotic device, they will charge you for producing them, but the problem is PWD do not have money. As I said earlier, most of them beg in the streets. Some of them used to work with crutches and orthotic device, but now you find them on wheelchairs, some are even crawling on the ground because there is no money for all these things”. FGD F, Participant 29, disability advocate, non-governmental organisation.

Participants described that importing assistive devices for PWD is tax-free, but there were still challenges. An NGO had trained carpenters and tailors to make rehabilitation equipment that was used across the country. They suggested that industry within the country should make assistive devices and small deposit fees for crutches to facilitate multiple persons use.

#### Lack of specialised services and rehabilitation personnel for complex rehabilitation needs

Participants expressed that multiple groups of PWD had complex healthcare and rehabilitation needs. They described rehabilitation services and domestic trained professionals such as occupational therapists, physical therapists, audiologists, and certified prosthetist/orthotist technicians were lacking in most districts. They feared the time they would retire, there would be an increase in the need for rehabilitation services, which would be a problem unless newly qualified professionals were trained.

“How many people do we have that are professionals and can handle eye problems? How many do we really have that can make prostheses for PWD? How many doctors do we really have that can perform spina bifida operations? There should be provisions to ensure Sierra Leoneans to the sector”. FGD G, Participant 35, leadership/policy expert, Governmental organisation,

Participants described specialised health and rehabilitation wards were lacking to treat persons with cerebral palsy, cerebral malaria, stroke, and spinal cord injuries. Participants expressed hope because a specialised unit on stroke and spinal cord injuries was under development, and improved internet infrastructure could increase the possibility to use more advanced assistive technologies. Participants expressed integrating rehabilitation services into regional hospitals had increased the access to rehabilitation services for a broader group of patients. Participants suggested greater teamwork between healthcare and rehabilitation professionals could lead to improved treatment and rehabilitation outcomes. Participants described that persons with visual impairments lacked assistive devices like canes, braille printers and computers and using health technology to assist PWD was not developed in-country. The lack of sign language interpreters caused communication barriers between healthcare and rehabilitation personnel and persons with hearing impairments. Rehabilitation personnel like prosthetist/orthotist technicians were not currently under a government scheme of service; however, this was under development and awaited validation. A new physiotherapy education had been launched in Sierra Leone, but participants feared that newly trained physiotherapists would leave the country. Participants suggest qualified rehabilitation personnel were needed to stop stigmatisation by sensitising communities and counselling in rural areas. Rehabilitation personnel cared for the services and expressed pride in working in rehabilitation for PWD.

“The problem in Sierra Leone is these multidisciplinary team. We do not have the surgeons, the orthopaedic surgeons. It should be a team that are doing the operation. The physiotherapist should be there, the prosthetist should be there so that they can guide you. The way you are doing the amputation, so that they can affect the way you prepare the prosthesis, but they are not really doing this in Sierra Leone”. FGD C, Participant 14, certified physiotherapist, governmental organisation.

#### Need for continuous education of new and current rehabilitation personnel

Participants agreed that strengthening rehabilitation services requires the education of additional rehabilitation professionals. Current rehabilitation personnel needed further training to provide better rehabilitation services. People often go abroad to become rehabilitation professionals as prosthetists and orthotist had no training possibilities domestically. Participants described there were no rehabilitation courses at the college of medicine; however, the first bachelor’s degree in physiotherapy opened in 2019. However, participants were worried that the physiotherapy school would not have enough qualified teachers or rehabilitation personnel to supervise students or the needed equipment to ensure high-quality education.

“There are people going for all sorts of courses in the medical schools. Some go for nurses, some go for dentistry, but people do not go for rehabilitation because, in the college of medicine, there is no curriculum for that. If we have professionals that could be trained, and the curriculum could be introduced into the educational sector of the country. I think that can enhance the continuous training of rehabilitation service providers in the country”. FGD D, Participant 17, certified occupational therapist, governmental organisation.

Participants suggested creating networks with other physiotherapy schools internationally was needed to exchange experiences and increase the quality of education. Furthermore, participants suggested approaching NGOs to fund educational opportunities for rehabilitation subjects.

“There is a need for a link, gaining experience from other physiotherapy schools. Some of our physiotherapist went to Tanzania and Kenya. If there is this link with other schools who are practising the modern approach and interventions, we can be capacitated”. FGD A, Participant 1, disability advocate, non-governmental organisation.

## Discussion

Stakeholders identified that barriers to accessing healthcare and rehabilitation services for PWD were financial constraints, long distances and inaccessible environments to the health and rehabilitation facilities, and negative attitudes of healthcare personnel. Rehabilitation and healthcare facilities lacked specialised services, educated personnel and materials to provide quality services for complex rehabilitation needs. PWD continued to face stigmatisation throughout life, and there was a need to build their capacity for greater inclusion in the Sierra Leonean society. The major findings from the stakeholders perspective correspond with the experiences of persons with physical disabilities accessing and using rehabilitation services in Sierra Leone [11].

Financial constraints for PWD were a barrier to accessing rehabilitation services, and this finding confirmed the results of several previous studies [19–22]. In Sierra Leone, the high costs and inability to pay for rehabilitation services were reported as challenging for PWD [11, 19]. In South Africa [22], Namibia, Sudan, and Malawi [20], financial constraints to pay for rehabilitation services was a major barrier for PWD to access rehabilitation services [20, 21]. Among the working-age population in Sierra Leone, of those with a disability, 58% were employed compared to 63% of persons without a disability. Higher self-employment rates were also reported in the disability group compared to those without disabilities [2]. In Sierra Leone, where PWD most commonly have low economic status, rehabilitation services need to be free or low cost. Therefore, rehabilitation programmes need to consider affordability for the persons they are intended for to ensure access and use of rehabilitation programmes.

Our results indicated long distances and inaccessible environments as barriers to accessing health and rehabilitation services for PWD. In Sierra Leone, 7 out of 10 persons with a disability live in rural communities, and people living in rural areas were poorer than those living in urban areas [2]. Previous studies in Sierra Leone reported [19] that 40% of people with lower-limb disability [19] and people with different types of disabilities [11] experienced barriers to accessing rehabilitation services due to long distances to rehabilitation facilities, lack of transportation, or not having someone to go with them. A household-based survey from Namibia, Sudan, South Africa and Malawi also identified transportation as a major barrier [20]. A qualitative study in South Africa [22] explored barriers and facilitators to accessing rehabilitation services for persons with lower-limb amputations, and distance and transportation obstacles were among the barriers identified. In a systematic review in low and middle-income countries, long distances were identified as a common barrier that PWD experienced in accessing rehabilitation facilities, as well as not knowing about the availability of rehabilitation services [21]. In South Africa, facilitators to accessing rehabilitation included a working referral system where the participants were referred to them, even though they were not initially aware of these services and increased self-motivation by the physiotherapist and family support [22]. To increase access to rehabilitation services in Sierra Leone and other low-income countries, transportation issues need to be considered, and rehabilitation facilities need to be available in several locations in combination with a referral system to provide increased access for persons living in rural areas and far from services.

Our results indicated a lack of governance and disjointed health and rehabilitation service delivery in Sierra Leone. Health and rehabilitation service delivery was fragmented and lacked specialised services, educated personnel and materials to provide quality services for basic and complex rehabilitation needs. The urgency to increase the capacity of the health and rehabilitation workforce has been brought to attention both internationally and in Sierra Leone [6, 23–25]. There is a pressing issue with a shortage of rehabilitation personnel; only a few qualified Sierra Leonean physiotherapists work in the country. Prosthetics and orthotic technicians wanted further education with rehabilitation, prosthetic and orthotic design to increase their capacity [26]. Sierra Leone need to allocate resource to start educating rehabilitation professionals either locally or in collaboration with neighbouring countries.

A systematic literature review [21] including 77 articles in low- and middle-income countries investigating access to rehabilitation services in particular: use of assistive devices, usage of healthcare services specialised in rehabilitation and consistency to treatment found that coverage of different types of rehabilitation for PWD was poor in many countries but also varied greatly within countries. The shortage of qualified rehabilitation personnel such as physiotherapists, occupational therapists [27], prosthetists, and orthotists [28] is a persisting barrier in Sierra Leone and many other African countries. Sierra Leone has in 2019 established its first bachelor’s physiotherapy programme.

One global response in low resource settings to the lack of rehabilitation personnel is CBR. CBR is now widely used across disciplines, mostly in low-income settings, to provide rehabilitation for PWD in their communities by community rehabilitation workers [1, 29–31]. Task-shifting, meaning shifting the responsibility or execution of certain tasks normally performed by highly qualified personnel to personnel with shorter education, is applied in CBR programmes. This approach has been widely used where qualified healthcare and rehabilitation personnel are lacking to expand coverages and improve access [6, 32]. Thus, CBR workers can contribute to facilitating inclusion for PWD in communities and their access to society. However, educated rehabilitation personnel are needed to provide high-quality, specialised rehabilitation services, including PWDs for on-the-job training to staff rehabilitation facilities, where CBR workers can refer PWD when necessary.

Rehabilitation services faced major constraints with low quality and unavailability of materials, insufficient equipment, and facilities, which led to the poor realisation of rehabilitation services needed by PWD. These findings confirm earlier studies [11, 26] where rehabilitation personnel in Sierra Leone indicated that the lack of materials and insufficient equipment was a major constraint and continue to be a persisting barrier to providing rehabilitation services [11, 26].

Negative attitudes of healthcare personnel towards PWD in Sierra Leone affects their access to health and rehabilitation services and corresponds to the results of a systematic literature review which including several low and middle-income countries where PWD experienced negative attitudes, lack of communication and lack of knowledge about disability issues from healthcare personnel [21]. A cross-sectional study conducted in Sierra Leone assessing access to reproductive health and healthcare for PWD found they had worse health and less access to healthcare facilities than people without disabilities [33].

PWD continued to face stigmatisation throughout life, and there is a need to build their capacity for greater inclusion in the Sierra Leonean society. These findings correspond with previous studies reporting a low awareness of disability issues among the society and rehabilitation personnel expressing that PWD was marginalised [11, 26, 34]. The marginalisation in Sierra Leone of PWD also became evident in a qualitative studies [35] using in-depth interviews with PWD. Results showed that PWD experienced negative attitudes from society and community. They reported that traditional beliefs such as PWD being the devil or being exposed to witchcraft were still prevalent. PWD struggle to get employment and education and reveal that having access to assistive devices positively impacts their lives, because they can do things, they otherwise could not do [11, 35]. Work to promote inclusive education enhancing the participation of PWD in education has started, but it needs to be scaled up. The community’s negative attitude towards PWD needs to be addressed. Increasing knowledge and awareness-raising, including the traditional stakeholders like priests, imams, and chiefs, together with the implementation of policies, could be one way forward. In Cameroon, sensitising the community about the abilities of PWD had a positive impact. It reduced the community’s negative perception of PWD [36]. Continuous work in increasing knowledge to change attitudes towards PWD is needed in Sierra Leone to facilitate increased inclusion in society.

The strength of this study is the inclusion of seven FGD, 37 participants generating a rich saturated data material. Participants in the FGDs representing a large variation of stakeholders working within the field of disability across several locations in Sierra Leone providing a good national coverage of the workforce within the field, however participants from the Kono district were missing. Limitations in the qualitative study design used has limited transferability [16] of the results to other contexts.

In conclusion, rehabilitation services need to be free or low cost to be affordable for the PWD in Sierra Leone. To increase access, transportation costs for PWD living in rural areas and far from rehabilitation services need to be covered. To scale up and ensure rehabilitation services increased numbers of locally trained qualified rehabilitation staff is needed. PWD continued to face stigmatisation throughout life, and there is a need to build their capacity and educate the public on disability issues for greater inclusion in society.

CBR programmes and workers can facilitate greater inclusion in the society for PWD, especially in remote rural areas. Still, rehabilitation centres with qualified rehabilitation personnel are needed for specialised services to which CBR workers can refer PWD to. Local actors need to take charge and renew efforts made by international organisations by providing trained rehabilitation staff and quality rehabilitation services in several geographical locations and develop a national referral system. In addition, continuous education about disability issues and inclusion is necessary for the public and healthcare personnel to reduce negative attitudes towards PWD.

## Data Availability

The datasets used and analysed in the current study are available from the corresponding author on reasonable request.

## Abbreviations

CBR: Community-based rehabilitation
CRPD: Convention on the Rights of Persons Living with Disabilities
FGD: Focus group discussions
NGOs: Non-governmental organisations
PWD: Persons with disabilities
